# Supplementary material for: F2BMF (M = B and Al) Molecules: A Matrix Infrared Spectra and Theoretical Calculations Investigation
Source: Molecules. 2023 Jan 5;28(2):554. doi: 10.3390/molecules28020554 (PMC9867038; doi:10.3390/molecules28020554)
Supplement: Supplementary file 1 [file molecules-28-00554-s001.zip › molecules-2017436-supplementary.pdf]

molecules
MDPI

Supplementary Materials

F_2BMF (M = B and Al) Molecules: A Matrix Infrared Spectra and Theoretical Calculations Investigation

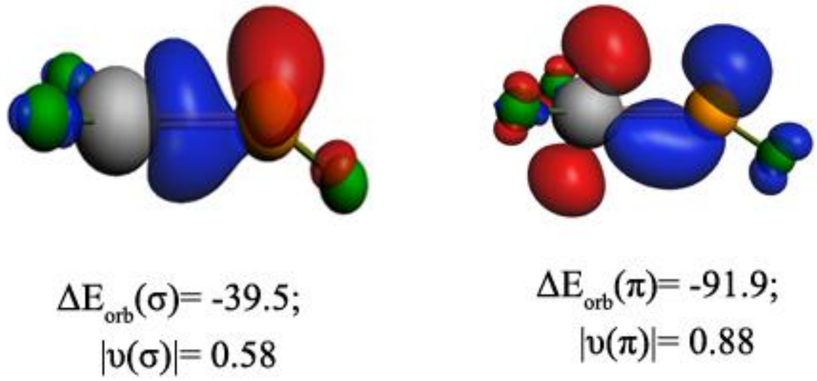

ΔE_{int}	ΔE_{Pauli}	ΔE_{elstat}	ΔE_{orb}	ΔE_{σ}	ΔE_{π}	ΔE_{orb}(rest)	ΔE_{dist}
-122.0	59.1	-46.8 (25.8%)	-134.7 (74.2%)	-39.5 (29.3%)	-91.9 (68.3%)	-3.3 (2.4%)	0. 39

Figure S1. Plot of the deformation densities Δϱ of the BF→AlF_2 σ donation and AlF_2→BF π back-donation in $FBAlF_2$ with the associated interaction energy ΔE_{orb} and charge eigenvalues $|\nu_n|$(in e). The charge flow is from red → blue.

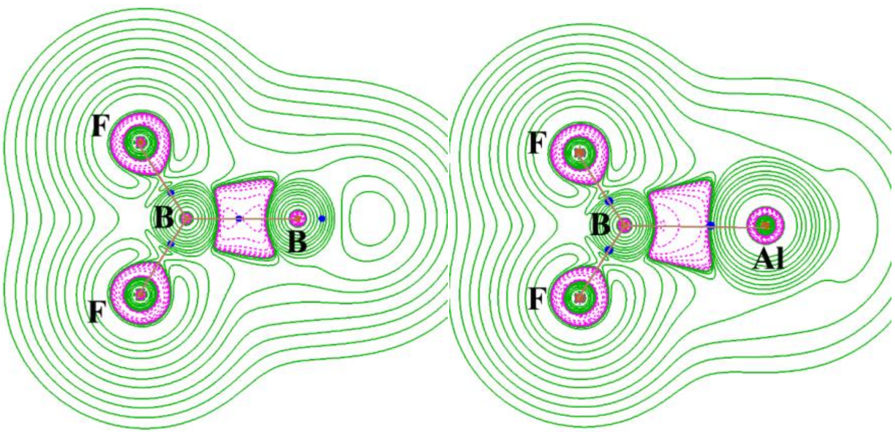

Figure S2. Contour line diagrams of the Laplacian of the electronic density of F_2BMF (M = B, Al). Red lines are in regions of the negative charge concentrations ($\nabla^2 \varrho(r) < 0$); Green lines are in regions of the positive charge depletion ($\nabla^2 \varrho(r) > 0$).

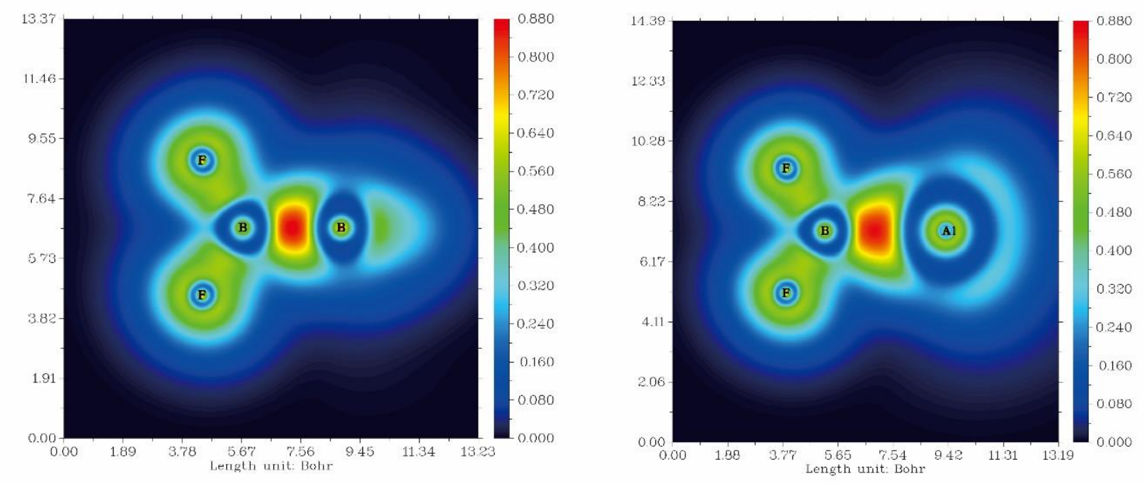

Figure S3. Color-filled maps of localized orbital locator of F_2BMF molecules (M = B, Al) at B3LYP/def2-TZVPP level.

Table S1. Compositions of Natural Bond Orbitals from NBO Analysis of F_2BMF molecules (M = B, Al).

Molecule	Bond	NBO	OCC
F_2BBF	B^1-B^4 σ	$34.50\%B^1(s^{0.29}p^{0.71})+65.50\%B^4(s^{0.66}p^{0.34})$	α 0.95
		$47.41\%B^1(s^{0.41}p^{0.59})+52.59\%B^4(s^{0.62}p^{0.38})$	β 0.99
	B^1-B^4 π	$34.13\%B^1(s^{0.15}p^{0.85})+66.87\%B^4(p)$	α 0.96
	F^1-B^1 σ	$17.42\%B^1(s^{0.28}p^{0.71}d^{0.01})+82.58\%F^1(s^{0.44}p^{0.56})$	α 1.00
		$17.42\%B^1(s^{0.29}p^{0.70}d^{0.01})+82.58\%F^2(s^{0.44}p^{0.56})$	β 1.00
	F^2- B^1σ	$17.42\%B^1(s^{0.28}p^{0.71}d^{0.01})+82.58\%F^1(s^{0.44}p^{0.56})$	α 1.00
		$17.42\%B^1(s^{0.29}p^{0.70}d^{0.01})+82.58\%F^2(s^{0.44}p^{0.56})$	β 1.00
	B^4-F^3 σ	$16.05\%B^4(s^{0.35}p^{0.65})+83.95\%F^3(s^{0.44}p^{0.56})$	α 1.00
		$15.97\%B^4(s^{0.36}p^{0.64})+84.03\%F^3(s^{0.45}p^{0.55})$	β 1.00
F_2BAlF	B-Al σ	$76.09\%B(s^{0.44}p^{0.56})+25.57\%Al(s^{0.16}p^{0.82}d^{0.02})$	α 0.99
		$50.50\%\ B(s^{0.39}p^{0.61})+49.50\%Al(s^{0.83}p^{0.17})$	β 0.99
	Al (LP)	$100\%Al(s^{0.75}p^{0.25})$	α 0.98
	F^1-B σ	$16.14\%B(s^{0.28}p^{0.71}d^{0.01})+83.86\%F^1(s^{0.46}p^{0.54})$	α 0.99
		$16.43\%B(s^{0.30}p^{0.69}d^{0.01}+83.57\%F^1(s^{0.45}p^{0.55})$	β 0.99
	F^2-B σ	$16.14\%B(s^{0.28}p^{0.71}d^{0.01})+83.86\%F^1(s^{0.46}p^{0.54})$	α 0.99
		$16.43\%B(s^{0.30}p^{0.69}d^{0.01}+83.57\%F^1(s^{0.45}p^{0.55})$	β 0.99

All the data are calculated by B3LYP hybrid density functional. LP denotes lone pair.

Table S2. Effective Bond Order Computed at B3LYP/def2-TZVPP level of F_2BMF molecules (M = B, Al).

Orbital	F_2BBF	F_2BAlF
a(σ_1)	0.952	0.989
a (σ_1*)	0.084	0.020
a (σ_2)	0.956	0.988
a (σ_2*)	0.117	0.042
a (π)	0.995	/
a (π*)	0.016	/
EBO	1.32	0.96
WBI	1.05	0.69

Table S3. Calculated Fundamental Frequencies of F_2BBF and F_2AlBF isotopomers in the Ground ^2A State.

Approximate description	Cal(int)[a]	Cal(int)[b]	Cal(int)[c]	Cal(int)[d]	Cal(int)[a]	Cal(int)[b]	Cal(int)[c]	Cal(int)[d]
		**$F_2{}^{11}B^{11}BF$**					**$F_2{}^{10}B^{11}BF$**	
**BBFantisymstr**	1458.1(196)	1491.2(52)	1461.7(50)	1466.2	1502.2(312)	1501.4(41)	1471.7(37)	1477.8
**BF_2antisymstr**	1425.9(204)	1332.3(294)	1286.7(265)	1376.3	1453.7(123)	1378.8(317)	1331.6(284)	1425.1
**BF_2symstr**	1261.1(370)	1188.9(465)	1150.9(427)	1213.6	1287.1(384)	1221.3(505)	1182.6(465)	1245.4
**BF_2scis**	762.0(4)	658.5(24)	640.0(18)	674.6	775.5(8)	663.9(22)	644.8(16)	683.5
**BF_2B def**	661.3(49)	570.9(19)	540.0(13)	605.9	675.6(48)	591.6(20)	560.2(14)	624.1
**BF_2 bend**	485.2(26)	401.0(1)	387.3(1)	414.1	488.0(27)	401.3(1)	387.6(1)	414.9
**FBB wag**	465.6(3)	366.5(0)	363.8(0)	335.5	466.7(3)	367.5(0.7)	364.7(0)	336.6
**BBF bend**	295.8(11)	188.3(15)	182.3(14)	194.3	296.7(11)	188.9(15)	187.8(14)	195.1
**BF_2BF def**	182.5(6)	125.2(2)	119.5(2)	115.5	183.1(6)	125.6(2.5)	119.9(2)	115.8
		**$F_2{}^{11}B^{10}BF$**					**$F_2{}^{10}B^{10}BF$**	
**BBFantisymstr**	1485.7(89)	1538.6(70)	1508.0(63)	1511.4	1512.1(201)	1546.8(56)	1516.6(49)	1520.8
**BF_2antisymstr**	1437.5(316)	1332.3(294)	1286.7(265)	1376.3	1478.2(228)	1378.9(324)	1331.6(284)	1425.1
**BF_2symstr**	1270.5(373)	1192.3(462)	1154.2(424)	1217.9	1301.1(399)	1226.3(505)	1187.3(463)	1251.9
**BF_2scis**	776.4(3)	663.8(25)	644.9(19)	683.1	789.0(7)	669.3(23)	649.8(17)	682.1
**BF_2B def**	661.5(3)	572.7(19）	542.1(13)	607.1	676.0(48)	593.0(20)	561.6(14)	624.7
**BF_2 bend**	493.7(27)	405.5(1)	391.3(1)	419.5	496.9(28)	405.9(0.98)	391.7(1)	420.5
**FBB wag**	480.8(4)	378.2(0)	375.7(0)	345.6	482.0(4)	379.2(0.1)	376.6 (0)	346.6
**BBF bend**	298.2(11)	192.6(16)	191.6(15)	197.8	299.2(11)	193.2(16)	192.3(15)	198.7
**BF_2BF def**	183.7(6)	125.9(2.5)	120.0(2)	116.3	184.4(6)	126.3(2.5)	120.5(2)	116.6
		**$F_2{}^{11}BAlF$**					**$F_2{}^{10}BAlF$**	
**BF_2antisymmstr**	1411.1(300)	1312.0(277)	1270.9(257)	1342.1	1460.4(321)	1357.4(299)	1314.9(275)	
**BF_2symstr**	1277.2(319)	1172.2(320)	1133.9(309)	1190.3	1317.8(347)	1208.2(378)	1168.8(336)	1227.4
**AlFstr**	831.0(117)	806.7(106)	780.1(98)	814.5	831.0(116)	806.8(106)	780.2(98)	814.6
**BF_2 scis**	624.5(15)	566.6(20)	549.5(17)	576.0	631.0(12)	572.5(18)	555.2(15)	582.0
**F_2BAlF def**	559.0(29)	466.8(32)	453.4(31)	472.9	580.9(33)	484.6(34)	470.4(34)	491.1
**F_2BAlF def**	338.3(30)	300.7(12)	290.7(8)	311.9	338.8(31)	301.6(13)	291.6(9)	312.8
**F_2BAl wag**	231.2(1)	162.1(0)	157.3(0)	166.7	232.5(1)	163.1(0)	158.2(0)	167.7
**BAlF bend**	156.7(15)	118.7(9)	114.3(9)	124.7	1567.0(15)	118.9(9)	114.5(7)	124.9
**F_2BAlF def**	99.9(8)	49.9(4)	52.9(4)	33.8	99.9(8)	49.9(4)	52.9(4)	33.8

The vibrational frequencies (cm^{-1}) and intensities (km/mol, in parentheses) are calculated using: [a] CAS(9e, 11o)/def2-TZVP basis set; [b] B3LYP/def2-TZVPP basis set.; [c] BPW91/def2-TZVPP basis set; [d]CCSD(T)/def2-TZVP(-f) basis set.

The value predicted by CASSCF method is 1425.9 (1453.7) and 1261.1(1287.1) cm^{-1} for the anti-symmetric and symmetric B-F stretching modes of $F_2{}^{11}B(^{10}B)$-^{11}BF molecule, which is overestimated by about 93.6 cm^{-1} (74.9 cm^{-1}) and 72.2 cm^{-1} (65.8 cm^{-1}) respectively. For $F_2{}^{11}B(^{10}B)$-^{10}BF molecule, the predicted value calculated by CASSCF is also overestimated by 98.0 cm^{-1} (107.6 cm^{-1}) and 69.7 cm^{-1} (59.5 cm^{-1}). Similarly, for the F_2BAlF molecule, the calculated BF_2 anti-symmetric and symmetric mode is at 1411.1 and 1277.2 cm^{-1} by CASSCF method, , being overestimated by about 129.5 cm^{-1} and 96.4 cm^{-1}respectively.
